# Evidence that NO/cGMP/PKG signalling cascade mediates endothelium dependent inhibition of IP_3_R mediated Ca^2+^ oscillations in myocytes and pericytes of ureteric microvascular network *in situ*

**DOI:** 10.1016/j.ceca.2015.08.006

**Published:** 2015-12

**Authors:** Lyudmyla Borysova, Theodor Burdyga

**Affiliations:** Department of Cellular and Molecular Physiology, Institute of Translational Medicine, University of Liverpool, Crown St, L8 7SS, UK

**Keywords:** Myocytes, Pericytes, Endothelium, Ca^2+^ signalling, Microvascular networks, Nitric oxide, cGMP, PKG

## Abstract

In ureteric microvessels the antagonistic relationship between Ca^2+^ signalling in endothelium and Ca^2+^ oscillations in myocytes and pericytes of arterioles and venules involves nitric oxide (NO), but the underlying mechanisms are not well understood. In the present study we investigated the effects of carbachol and NO donor SNAP on Ca^2+^ signalling and vasomotor responses of arterioles and venules in intact urteric microvascular network *in situ* using confocal microscopy. Vasomotor responses of arterioles and venules induced by AVP correlated with the occurrence of Ca^2+^ oscillations in the myocytes and pericytes and were not abolished by the removal of Ca^2+^ from extracellular fluid. Carbachol-induced rise of intracellular Ca^2+^ in endothelium was accompanied by the termination of the Ca^2+^ oscillations in myocytes and pericytes. This carbachol-induced inhibitory effect on Ca^2+^ oscillations in myocytes and pericytes was reversed by ODQ, an inhibitor of soluble guanylyl cyclase (sGC) and by Rp-8-pCPT-cGMPS, an inhibitor of protein kinase G (PKG). Ca^2+^ oscillations in myocytes and pericytes were also effectively blocked by NO donor SNAP. An Inhibitory effect of SNAP was markedly enhanced by zaprinast, a selective inhibitor of cGMP-specific phosphodiesterase-5, and reversed by sGC inhibitor, ODQ and PKG inhibitor, Rp-8-pCPT-cGMPS. The cGMP analogue and selective PKG activator 8pCPT-cGMP also induced inhibition of the AVP-induced Ca^2+^ oscillations in myocytes and pericytes. SNAP had no effects on Ca^2+^ oscillations induced by caffeine in distributing arcade arterioles. Consequently, we conclude that NO- mediated inhibition of Ca^2+^ oscillations in myocytes and pericytes predominantly recruits the cGMP/PKG dependent pathway. The inhibitory effect of NO/cGMP/PKG cascade is associated with suppressed Ca^2+^ release from the SR of myocytes and pericytes selectively *via* the inositol triphosphate receptor (IP_3_R) channels.

## Highlights

• Endothelium-dependent inhibition of Ca^2+^ oscillations in myocytes and pericytes was reversed by ODQ, an inhibitor of soluble guanylyl cyclase (sGC).
• Selective PKG inhibitor Rp-8-pCPT-cGMPS, reversed endothelium- dependent termination of agonist-induced Ca^2+^ oscillations in myocytes and pericytes.
• Selective PKG activator 8pCPT-cGMP induced inhibition of the agonist-induced Ca^2+^ oscillations in myocytes and pericytes.
• Inhibitory effect of SNAP was markedly enhanced by zaprinast.
• Inhibitory effect of NO/cGMP/PKG cascade is associated with suppressed Ca^2+^ release *via* IP_3_Rs of myocytes and pericytes.

## Graphical abstract

**Figure:**
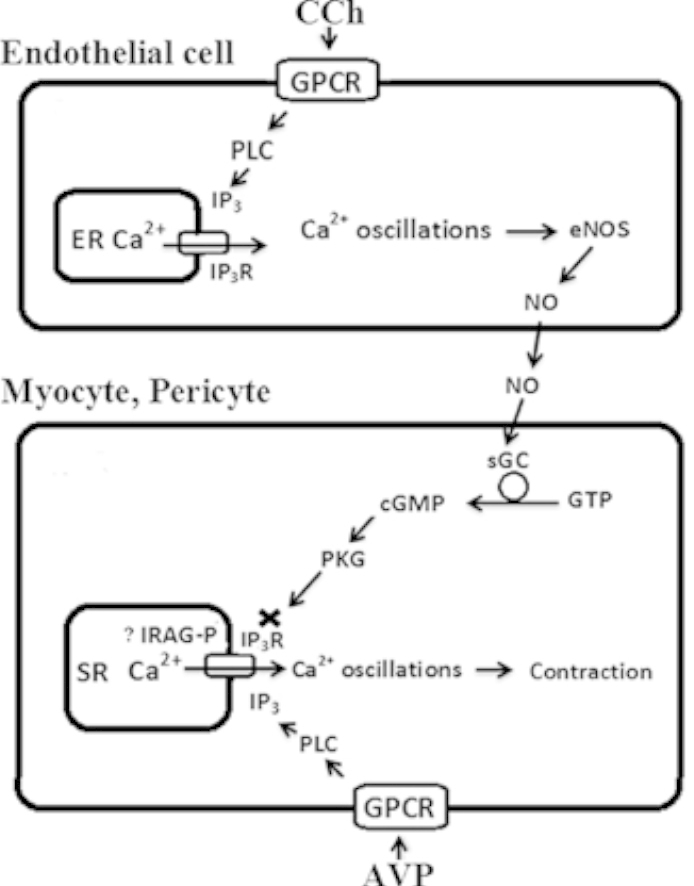

## 1 Introduction

An endothelial cell (EC) Ca^2+^ signalling is implicated in regulation of the arteriolar and venular microvascular tone and involves antagonistic relationship between Ca^2+^ signals in endothelial and media cells (myocytes and pericytes) [1], [2], [3], [4], [5], [6]. For example, dilation of ureteric arterioles and venules [6] or hamster cheek pouch arterioles [7] in response to the endothelium-dependent vasodilator carbachol (CCh) or acetylcholine (ACh) is associated with an increase in ECs Ca^2+^ signalling which terminates agonist induced Ca^2+^ signals in smooth muscle cells and pericytes. In myocytes and pericytes of ureteric microvessels the antagonistic relationship between Ca^2+^ signalling in endothelium and media cells is NO-mediated [6]. An increase in the intracellular concentration of free Ca^2+^ ([Ca^2+^]_i_) in ECs activates Ca^2+^/calmodulin-dependent constitutive endothelial NO synthase (eNOS) to generate NO, which diffuses to the adjacent myocytes or pericytes and binds to a prosthetic group on soluble gyanylyl cyclase (sGC). Stimulated sGC catalyses synthesis of the second messenger cGMP, which in turn activates cGMP-dependent PKG and/or other effector proteins, including ion channels, pumps, and phosphodiesterases (PDEs) [8]. Impaired NO and cGMP signalling have been implicated in the pathogenesis of cardiovascular disease and atherosclerosis [9], [10], [11]. In large blood vessels ACh-induced dilations are mainly mediated by NO/cGMP/cGKI pathway [12], [13]. The importance of NO and cGMP for the regulation of vascular tone and blood pressure has been recently strengthened by the observation that cGMP, eNOS, or cGKI deficiency in mice lead to pathological changes in vascular wall and hypertension development [12], [14], [15]. Although, the involvement of the sGC/cGMP pathway in the pharmacological actions of NO is widely accepted, there are data suggesting that alternative NO mediated cGMP-independent pathways also exist. It is accepted that NO- induced vasodilation can be classified as cGMP-dependent as long as it is completely inhibited by the selective blocker of soluble guanylate cyclase, ODQ (1H-[1,2,4]oxadiazole[4,3-a]quinoxalin-1-one) [16], [17], [18]. However, in some blood vessels a lack of ODQ effect on NO-induced inhibition of Ca^2+^ signalling and tone was reported and contribution of cGMP-independent mechanisms was suggested [18], [19], [20], [21], [22], [23], [24]. We hypothesized that in myocytes and pericytes of ureteric microvessels, NO activates a cGMP/PKG-dependent pathway. To test this hypothesis we examined the effects of putative pharmacological modulators of NO/cGMP/PKG pathway on the endothelium- mediated inhibition of AVP-induced Ca^2+^ oscillations in myocytes and pericytes of ureteric microvascular networks in situ in the absence and the presence of external Ca^2+^. To achieve this goal we used our unique methodology developed back in 2003 [25] of real time confocal imaging of intact microvascular networks *in situ*, which allows to monitor simultaneously an intracellular [Ca^2+^] in myocytes, pericytes and endothelial cells and correlate it with vasomotor activity [6], [26].

## 2 Methods

### 2.1 Animals and ureteric tissue samples

The experiments were performed on Wistar rats of both sexes (3–4 months old). Rats were humanely killed using CO_2_ anaesthesia followed by cervical dislocation, in accordance with UK legislation. Whole ureters were dissected, carefully cleaned of connective and paraureteric adipose tissue using fine curved scissors and keeping the sharp edges away from the tissue to avoid physical damage of the ureteric bundles.

### 2.2 Calcium and diameter measurements

For Ca^2+^ measurements ureters were cut into small segments (4–5 mm long) and loaded with Fluo-4. Strips of ureter were placed in a plastic cuvette containing 1 ml of HEPES-buffered Krebs solution including 15 μM-fluo 4-AM dissolved in dimethyl sulphoxide premixed with Pluronic F127 (final concentration of 0.01%). Loading was performed at 23 °C for 3 h with the cuvettes wrapped in black tape and rotated at 30 rpm. Tissue samples were then removed from the loading medium and placed in normal Krebs solution for at least 30 min to allow cleavage of fluo 4-AM to fluo 4 by intracellular esterases. Fluo-4 loaded segments of ureter were transferred to a custom-made perfusion chamber mounted on the stage of inverted Olympus microscope. Superfusion of the ureteric segments in the chamber was performed by applying a positive pressure valve controlled flow of solution *via* a 1 mm diameter tip attached to a 3-d mechanical manipulator (Narishige, Japan) which allowed positioning the superfusion tip in a desired region of the chamber. Solution was removed by suction at the other end of the chamber. All experiments were performed at 30 °C. We used a Nipkow disc, confocal microscope [6], [25], [26] (Ultraview, PerkinElmer), connected to an iXon cooled charge-coupled device camera (Andor Technology, UK). Andor Technology iQ or iQ2 data acquisition software was used for 2- and 3-dimensional confocal imaging of ureteric microvascular networks *in situ*. Images were collected at 33–66 frames per second using a ×60 water objective (NA 1.20) for spatial resolution or dry (×10, NA 0.42; ×20, 0.70 NA) for a larger field of view. To measure elemental events and Ca^2+^ waves in myocytes and pericytes, tangential sections were used, whereas radial sections through the centre of the microvessel were used to measure Ca^2+^ events in myocytes, pericytes, endothelial cells and changes in vessel diameter. Mechanical activity of individual smooth muscle cells was tracked by putting the region of interest close to the edge of the contracting cell. It was possible to correlate Ca^2+^ signalling with contraction of individual myocytes and pericytes in both radial and tangential sections.

### 2.3 Solutions

Physiological saline of the following composition was used (mM): NaCl 120.4, KCl 5.9, MgSO_4_ 1.2, CaCl_2_ 2, glucose 11.5, and HEPES 11. The Ca^2+^-free solutions contained 2 mM EGTA. [Arg^8^]-Vasopressin acetate salt (AVP), Carbachol (CCh), Caffeine, Zaprinast (ZAP), 1H-[1,2,4]Oxadiazolo[4,3-a]quinoxalin-1-one (ODQ), Rp-8-pCPT-cGMPS sodium salt, 8pCPT-cGMP, S-nitroso-N-acetyl-DL-penicillamine (SNAP) were from Sigma. Fluo-4 acetoxymethyl ester was from Molecular Probes, Life Technologies, UK.

[Arg^8^]-Vasopressin acetate salt, Carbachol, Caffeine, Rp-8-pCPT-cGMPS sodium salt, 8pCPT-cGMP were dissolved in water; S-nitroso-N-acetyl-DL-penicillamine (SNAP), 1H-[1,2,4]Oxadiazolo[4,3-a]quinoxalin-1-one (ODQ), Zaprinast in DMSO.

### 2.4 Statistics

A paired Student's *t* test was used to test for significant differences between means. All statistical values are expressed as mean ± SEM.

## 3 Results

### 3.1 Effect of sGC inhibition

**Fig. 1:**
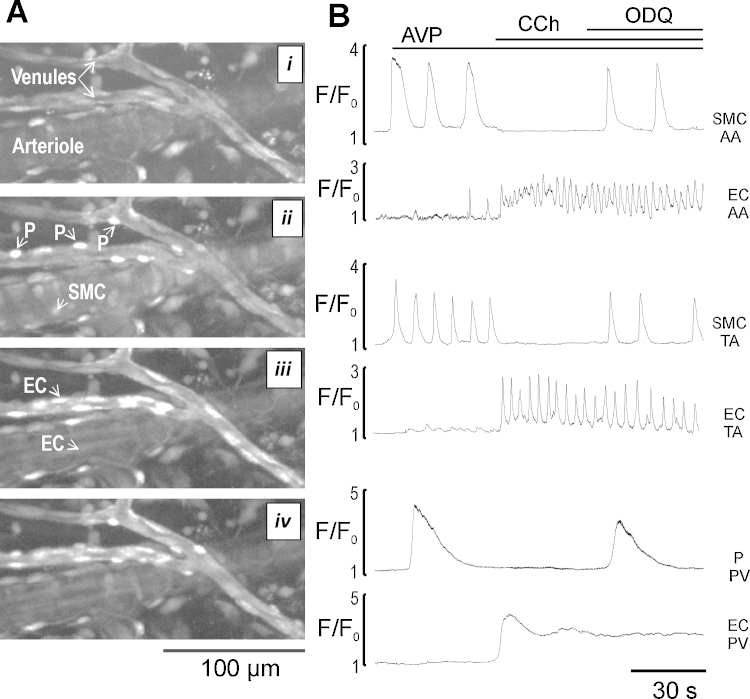
The reversal of an endothelium- dependent inhibition of AVP-induced Ca^2+^ oscillations in media cells (myocytes and pericytes) by sGC inhibitor, ODQ. (A) Images showing segments of ureteric arterioles and venules in radial section at rest (i), in the presence of AVP (5 nM) (ii), AVP and CCh (2 μM) (iii), and AVP + CCh + ODQ (25 μM) (iv). SMC – smooth muscle cells, EC – endothelial cells, P – pericytes respectively. (B) Ca^2+^ traces showing changes in intracellular Ca^2+^ in single endothelial (bottom trace) and media cells (top trace) of the arcade arterioles (AA), transverse arterioles (TA) and postcapillary venules (PV) at rest and during sequential application of AVP, AVP + CCh, and AVP + CCh + ODQ (*n* = 7).

**Fig. 2:**
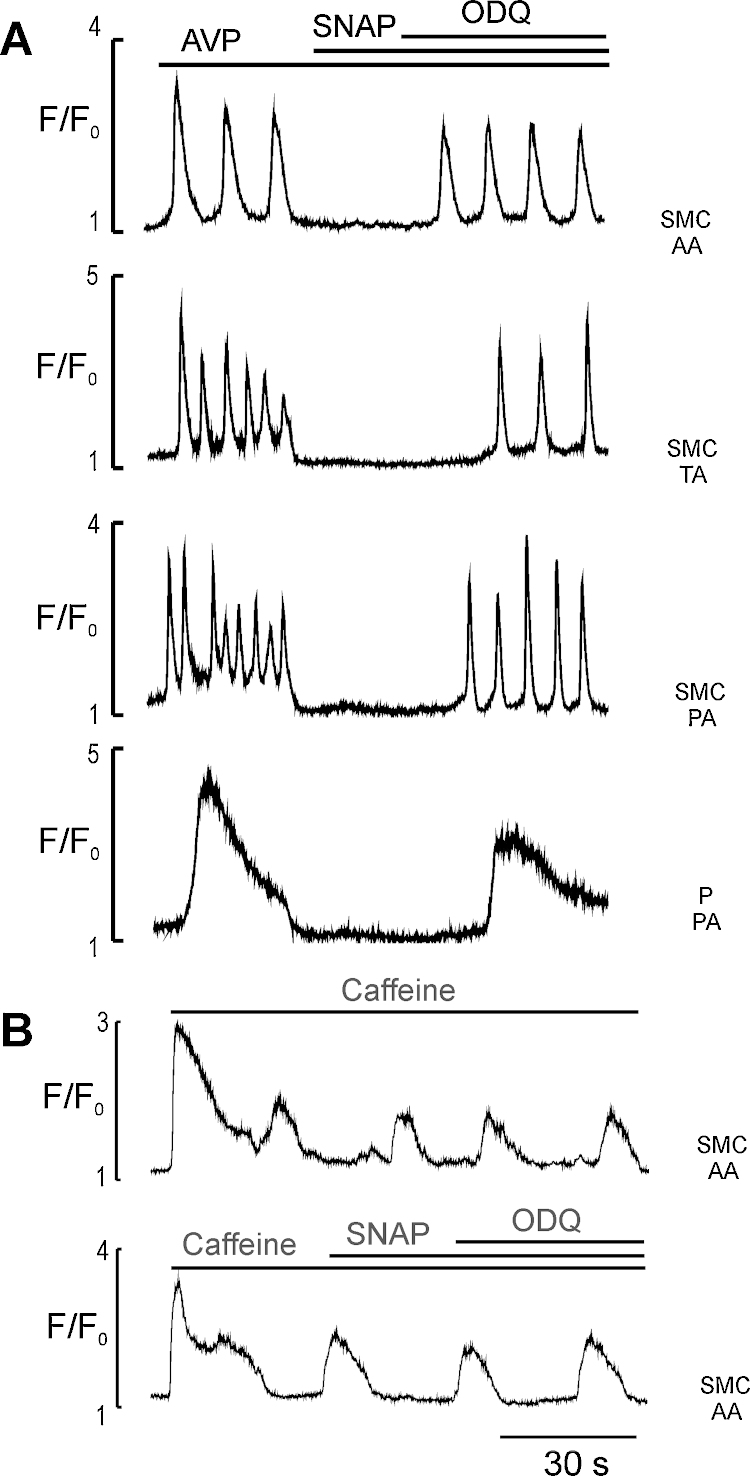
The effects of sGC inhibitor ODQ on AVP- and caffeine-induced Ca^2+^ oscillations in media cells in the presence of SNAP. (A) Ca^2+^ traces showing ODQ (25 μM)-induced reversal of an inhibitory effect of SNAP (10 μM) on AVP (5 nM)-induced Ca^2+^ oscillations in myocytes of AA, TA, precapillary arteriole (PA) and precapillary pericytes of PA (*n* = 7; (B) Ca^2+^ traces showing the lack of SNAP and ODQ effects on caffeine (1 mM)-induced Ca^2+^ oscillations in myocytes of AA in (*n* = 7).

To evaluate whether inhibition of AVP (5 nM)-induced Ca^2+^ oscillations in myocytes and pericytes of arteriolar and venular microvessels by CCh or SNAP is cGMP-dependent, we examined the effect of ODQ (25 μM), a selective inhibitor of NO-binding site on sGC, in the presence and absence of external Ca^2+^. Both myocytes and pericytes retain their ability to generate Ca^2+^ signalling and tone in the absence of an external Ca^2+^ [6], [26]. In these experiments we found that termination of sustained AVP-induced Ca^2+^ oscillations and vasomotor activity in myocytes and pericytes was associated with CCh (2 μM)-induced Ca^2+^ transient in endothelium and was reversed by ODQ which had no effect on Ca^2+^ signalling in endothelium or myocytes on its own (*n* = 7, Fig. 1A and B, Supplementary Videos 1 and 2). Furthermore, ODQ reversed an inhibitory action of SNAP (10 μM) on AVP-induced Ca^2+^ oscillations in myocytes and pericytes of arterioles and venules, suggesting an involvement of cGMP/PKG-dependent pathway (*n* = 7, Fig. 2). Similar results have been obtained in the absence of an external Ca^2+^. Fig. 2B also shows that SNAP had no effect on Ca^2+^ oscillations induced by 1 mM caffeine in myocytes of arcade arterioles – the only microvessels in ureteric microvascular network which express functional RyRs channels [6], suggesting that Ca^2+^ release mediated by RyRs channels was not affected by NO/cGMP/PKG cascade (*n* = 7).

### 3.2 Effects of PKG inhibition

**Fig. 3:**
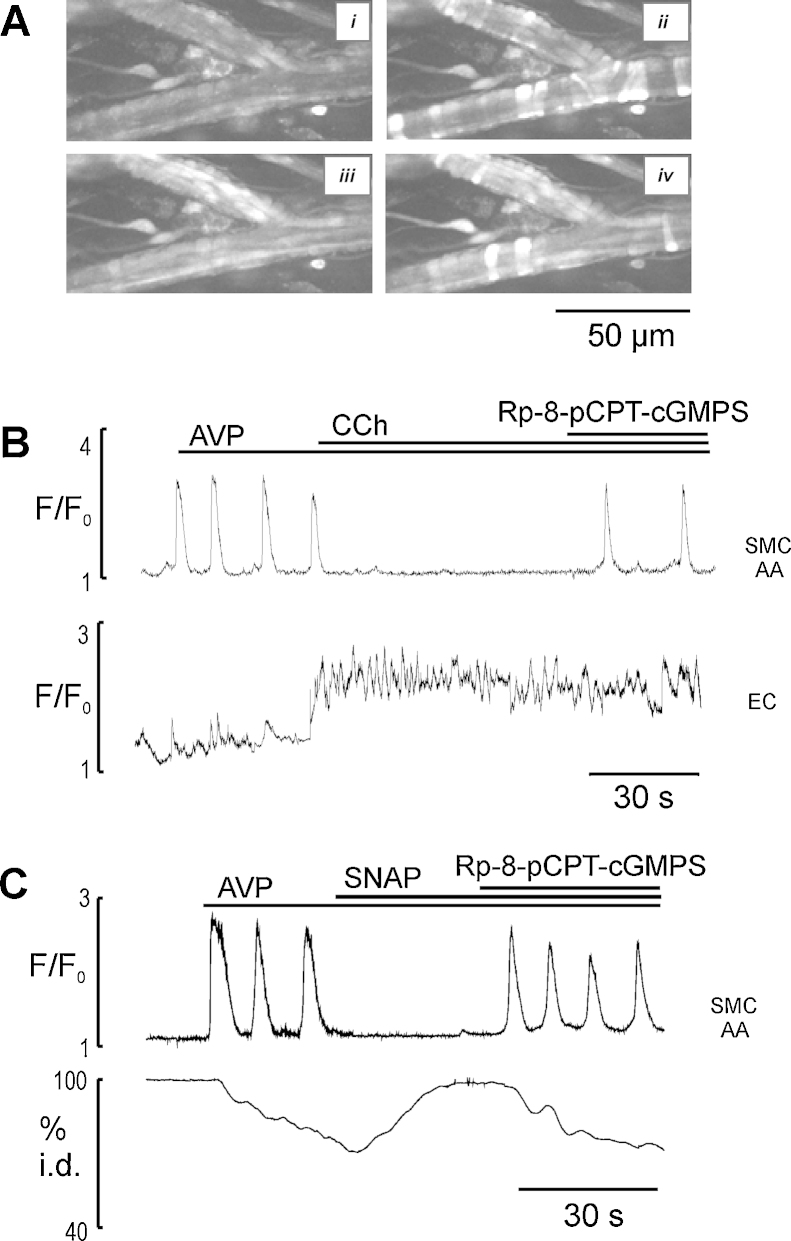
The reversal of inhibitory effects of CCh and SNAP on AVP-induced Ca^2+^ oscillations in myocytes of AA by PKG inhibitor Rp-8-pCPT-cGMPS in Ca^2+^-free media. (A) Images showing segments of ureteric AA in radial section at rest (i), in the presence of AVP (5 nM) (ii), AVP + CCh (2 μM) (iii), and AVP + CCh + Rp-8- pCPT-cGMPS (20 μM) (iv). (B) Ca^2+^ traces showing that Rp-8-pCPT-cGMPS reversed CCh-induced inhibition of AVP-activated Ca^2+^ oscillations in myocytes of AA (*n* = 7). (C) Ca^2+^ signal (top trace) and diameter change (bottom trace) showing that Rp-8- pCPT-cGMPS reversed SNAP (10 μM)-induced inhibition of AVP-activated Ca^2+^ oscillations in myocytes of AA (*n* = 7). Note that an endothelium-dependent inhibition of AVP-induced Ca^2+^ oscillations was performed in Ca^2+^ free media (preliminary arteriolar networks were exposed to Ca^2+^-free media with 2 mM EGTA for 3 min) suggesting Ca^2+^ entry is not important for eNOS activation in ureteric microvessels.

Since the above results suggest that endothelial cell Ca^2+^ signalling and NO donor SNAP inhibit Ca^2+^ oscillations in myocytes and pericytes, acting through NO/cGMP-dependent mechanism, we further investigated whether PKG mediates CCh- and NO-induced inhibitory effect on Ca^2+^ oscillations and tone in myocytes and pericytes. We found that a selective PKG inhibitor Rp-8-pCPT-cGMPS (20 μM) reversed the inhibitory effects of both CCh and SNAP on AVP - induced Ca^2+^ oscillations and tone in myocytes and pericytes of all sections of arterioles and venules in the presence or absence of an external Ca^2+^ (*n* = 7, Fig. 3 and Supplementary Video 3). These results suggest that PKG is required for NO-induced inhibition of Ca^2+^ oscillations mediated by IP_3_R channels in ureteric arteriolar myocytes and venular pericytes (data not shown).

### 3.3 Effect of PKG activation

**Fig. 4:**
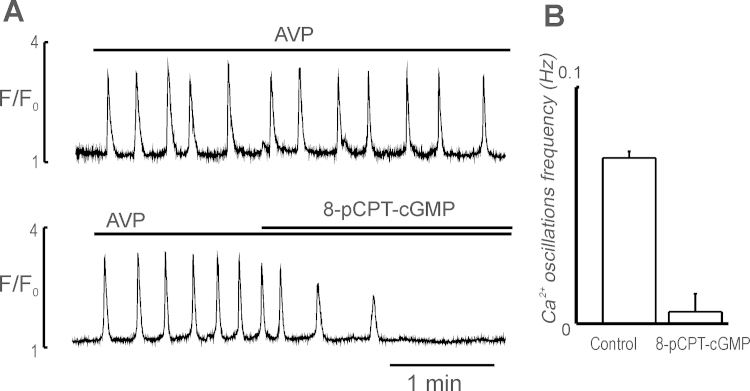
Inhibition of AVP-induced Ca^2+^ oscillations in myocytes of AA by cGMP analogue 8pCPT-cGMP. (A) Ca^2+^ oscillations induced by 5 nM AVP in myocytes of AA in the absence (top trace) and presence of 8pCPT-cGMP (100 μM) (bottom trace); (B) effect of 8pCPT-cGMP (100 μM) on the frequency of AVP-induced Ca^2+^ oscillations measured before (control) and 5 min after its application (*n* = 7).

To support the idea that SNAP effects on Ca^2+^ oscillations were mediated by PKG, we studied the effect of the potent PKG activator cGMP analogue 8pCPT-cGMP on the AVP induced Ca^2+^ oscillations in myocytes and pericytes of ureteric microvessels. This cGMP analogue is permeable to cell membranes, resistant to hydrolysis by PDEs, and selective activator of cGMP-dependent PKG. 8pCPT-cGMP (100 μM) caused time-dependent decrease in the amplitude and frequency of Ca^2+^ oscillations from 0.07 ± 0.002 Hz to 0.005 ± 0.001 Hz in 60% and completely ceased within 2–5 min in 40% of myocytes observed (*n* = 7, Fig. 4). These results suggest that raising cGMP targets PKG and induce inhibition of AVP-induced Ca^2+^ oscillations in myocytes and pericytes, leading to vasodilation.

### 3.4 Effect of PDE5 blocker zaprinast

**Fig. 5:**
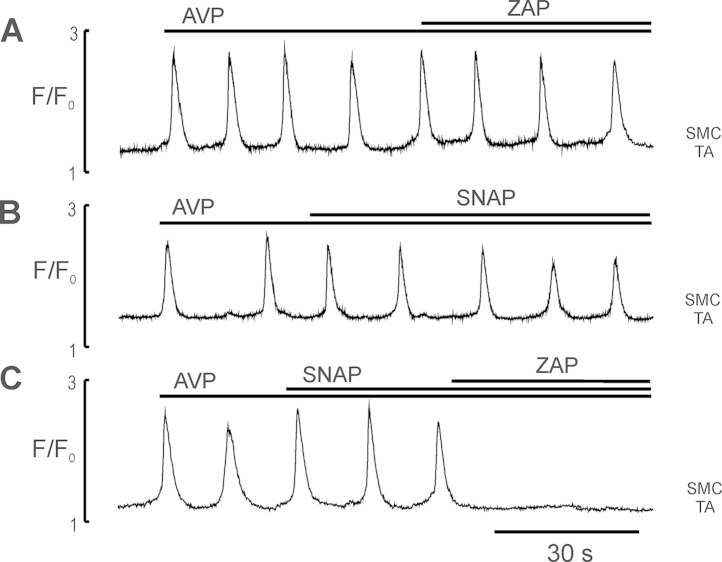
The effect of PDE5 inhibitor ZAP on AVP-induced Ca^2+^ oscillations in myocytes of TA in the absence and presence of the subthreshold concentration of SNAP. (A and B) Ca^2+^ traces showing no effect of ZAP (20 μM) and SNAP (10 nM) on the AVP (5 nM)-induced Ca^2+^ oscillations in single myocytes of AA, respectively; (C) Ca^2+^ trace showing that SNAP in subthreshold concentration completely inhibited AVP-induced Ca^2+^ oscillations in myocytes of AA in the presence of ZAP (*n* = 5). The data indicate that suppression of PDE5 significantly increased an inhibitory potency of SNAP.

Since NO action is mediated by second messenger cGMP with a short life span, we have examined how cGMP catabolism reduction by PDE-5-selective inhibitor zaprinast (ZAP) affected NO - sensitive Ca^2+^ oscillations in myocytes and pericytes of ureteric microvessels. In these experiments the microvessels were given a subthreshold concentration of SNAP (10–50 nM) in which it had no inhibitory effect on AVP induced Ca^2+^ oscillations (*n* = 5, Fig. 5B) and tone. Fig. 5A shows that ZAP alone had little or no effect on AVP-induced Ca^2+^ oscillations in myocytes and pericytes. However, combined action of SNAP (10 nM) and ZAP (20 μM) completely terminated AVP-induced Ca^2+^ oscillations in myocytes (*n* = 5, Fig. 5C) and pericytes of all sections of arteriolar and venular networks (data not shown). These data suggest that ZAP enhanced SNAP-mediated inhibition of AVP-induced Ca^2+^ oscillations of ureteric arterioles by supressing the cGMP-dependent PDE activity.

## 4 Discussion

In this study by combining confocal imaging with pharmacological analysis we investigated a possible role of NO/cGMP/PKG signalling cascade in endothelium- mediated Ca^2+^ dependent inhibition of agonist-induced Ca^2+^ oscillations and vasomotor responses in myocytes and pericytes of ureteric microvessels *in situ*. Previously, we have shown that in myocytes and pericytes of ureteric microvascular network agonist induced Ca^2+^ signalling and vasomotor responses are selectively mediated by IP_3_R channels dependent mainly on Ca^2+^ release from the SR. Similar data have been obtained on smooth muscle cells of intrapulmonary arterioles [27]. In addition, we have shown that activation of endothelial Ca^2+^ signalling by CCh (or bradykinin) terminated agonist-induced Ca^2+^ oscillations and vasomotor responses in the presence and absence of an external Ca^2+^. Furthermore, we have shown that antagonistic relationship between Ca^2+^ signalling in endothelium and media cells (myocytes and pericytes) was mainly NO-dependent. Here, we present experimental evidence suggesting that the inhibition of AVP-induced Ca^2+^ oscillations and vasomotor responses in myocytes and pericytes by CCh-induced endothelial Ca^2+^ signals employs NO/cGMP/PKG-dependent signalling cascade.

First, the inhibitory effects of endothelial cell Ca^2+^ signalling and NO donor SNAP on Ca^2+^ oscillations of myocytes and pericytes were quickly reversed by ODQ, a specific inhibitor of sGC that is activated by NO and synthesizes cGMP [16], [17], [18], and Rp-8-pCPT-cGMPS, a specific inhibitor of PKG activity [28]. Second, the membrane-permeable cGMP analogue 8pCPT-cGMP, which is a selective activator of cGMP-dependent PKG, inhibited Ca^2+^ oscillations induced by agonists in myocytes and pericytes. Third, in the presence of zaprinast, a selective inhibitor of cGMP-specific PDE-5 [28], the inhibitory effect of SNAP was amplified more than 10 times. Collectively these data suggest that cGMP- mediated mechanism is playing a key role in endothelium-dependent inhibition of Ca^2+^ oscillations and vasomotor responses in myocytes and pericytes of ureteric microvascular networks *in situ*. Our data are in a good agreement with the data obtained on vascular (rat tail artery) [29] and non-vascular (smooth muscle of airways) [28], [30] tissues.

**Fig. 6:**
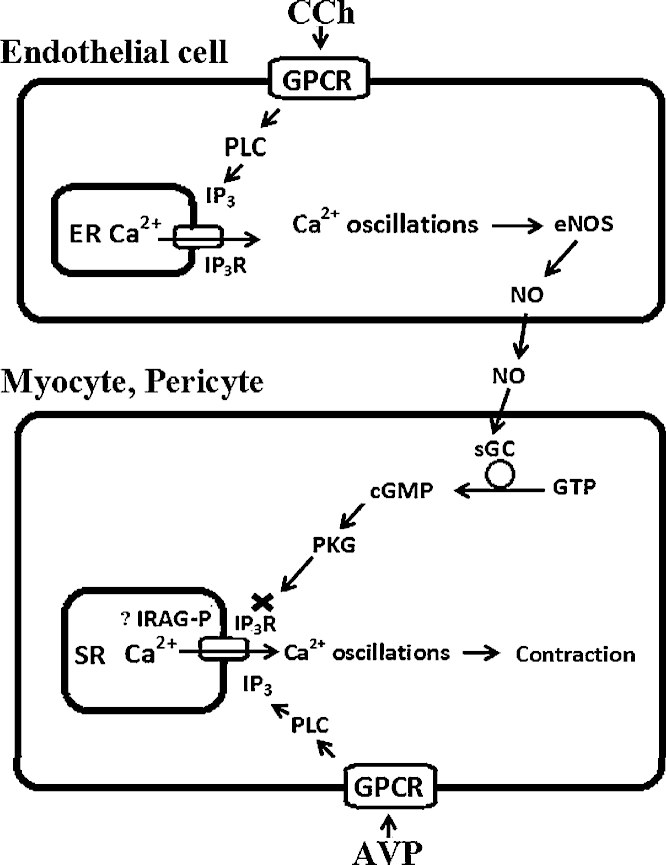
Schematic diagram showing signalling pathways involved in antagonistic relationship between Ca^2+^ signalling in endothelial and media (myocytes and pericytes) cells in ureteric microvessels. Agonists, such as CCh in endothelial cells (ECs) and AVP in myocytes and pericytes bind to their specific G protein-coupled membrane receptors (GPCR) and activate phospholipase C to synthesize IP_3_. IP_3_, in turn, activates IP_3_Rs to release Ca^2+^ from the ER/SR, inducing Ca^2+^ oscillations. Ca^2+^ oscillations in ECs activate eNOS to generate NO, and in myocytes and pericytes-myosin light chain kinase (MLCK) to trigger vasoconstriction. NO induces inhibition of Ca^2+^ oscillations in myocytes and pericytes by activating sGC to synthesize cGMP from GTP. The elevation of cGMP activates PKG, which inhibits the IP_3_R-mediated Ca^2+^ release (possibly *via* IRAG phosphorylation), resulting in termination of Ca^2+^ oscillations in media cells.

In addition, our data also indicate that NO had no effects on caffeine- induced Ca^2+^ oscillations in myocytes of distributing arcade arterioles where functional RyRs have been identified [6]. NO did not influence the magnitude of the Ca^2+^ release induced by caffeine. This result indicates that in myocytes of arcade arterioles, the capacity or Ca^2+^ stores content was not altered significantly and that the RyRs remained unaffected by NO, which agrees with the previous data [24], [28]. Collectively, these results suggest that NO, and the resulting increase in cGMP and PKG activity, decreased the frequency of agonist-induced Ca^2+^ oscillations by reducing the activity of the IP_3_Rs. The molecular mechanism by which NO/cGMP/PKG signalling cascade inhibited the IP_3_R was not investigated here, but an IP_3_R-associated cGMP kinase substrate protein called IRAG has been identified in some smooth muscle cells [31], [32]. This protein is associated with the IP_3_R, is phosphorylated by PKG, and upon phosphorylation it blocks IP_3_R activation by IP_3_ and Ca^2+^; this could be the major mechanism by which the NO/cGMP/PKG cascade reduces [Ca^2+^]_i_ and vascular tone [15] (Fig. 6).

Finally, we conclude that endothelial cell Ca^2+^ signalling terminates Ca^2+^ oscillations in myocytes and pericytes of ureteric microvessels by activation of NO/cGMP/PKG signalling cascade, which results in an inhibition of IP_3_R- mediated Ca^2+^ mobilization from the SR.

## Conflict of interest

The authors confirm that there are no conflicts of interest.
