# Supplementary figures and images for: Evidence that NO/cGMP/PKG signalling cascade mediates endothelium dependent inhibition of IP3R mediated Ca2+ oscillations in myocytes and pericytes of ureteric microvascular network in situ
Source: Cell Calcium. 2015 Dec;58(6):535–40. doi: 10.1016/j.ceca.2015.08.006 (PMC4655834; doi:10.1016/j.ceca.2015.08.006)

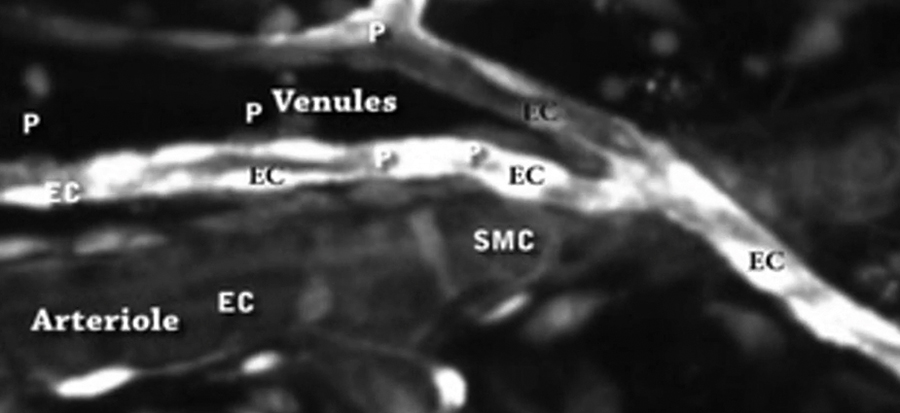

Supplement: Supplementary file 1 [file mmc1.jpg]

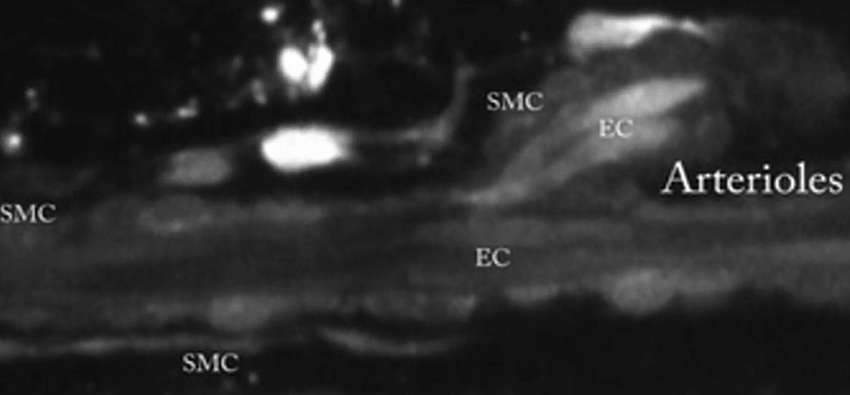

Supplement: Supplementary file 2 [file mmc2.jpg]

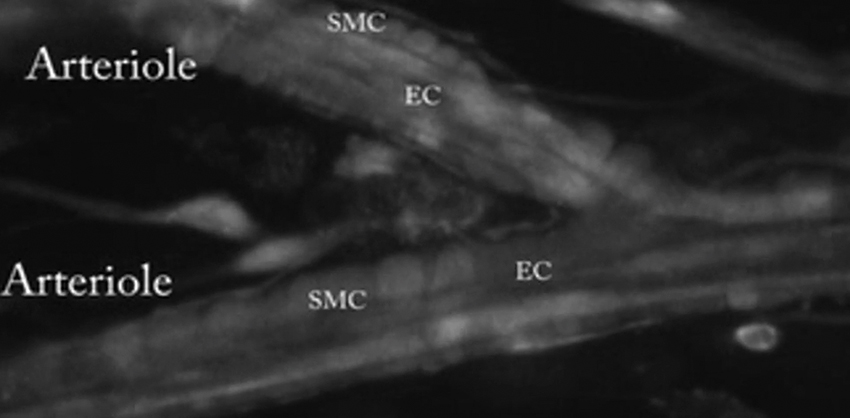

Supplement: Supplementary file 3 [file mmc3.jpg]
